# pH homeostasis links the nutrient sensing PKA/TORC1/Sch9 ménage-à-trois to stress tolerance and longevity

**DOI:** 10.15698/mic2018.03.618

**Published:** 2018-01-12

**Authors:** Marie-Anne Deprez, Elja Eskes, Tobias Wilms, Paula Ludovico, Joris Winderickx

**Affiliations:** 1 Functional Biology, KU Leuven, Leuven, Belgium.; 2 Life and Health Sciences Research Institute (ICVS), School of Medicine, University of Minho, Braga, Portugal; ICVS/3B’s - PT Government Associate Laboratory, Braga/Guimarães, Portugal.

**Keywords:** yeast, ageing, longevity, pH, V-ATPase, Pma1, PKA, TORC1, Sch9

## Abstract

The plasma membrane H^+^-ATPase Pma1 and the vacuolar V-ATPase act in close harmony to tightly control pH homeostasis, which is essential for a vast number of physiological processes. As these main two regulators of pH are responsive to the nutritional status of the cell, it seems evident that pH homeostasis acts in conjunction with nutrient-induced signalling pathways. Indeed, both PKA and the TORC1-Sch9 axis influence the proton pumping activity of the V-ATPase and possibly also of Pma1. In addition, it recently became clear that the proton acts as a second messenger to signal glucose availability via the V-ATPase to PKA and TORC1-Sch9. Given the prominent role of nutrient signalling in longevity, it is not surprising that pH homeostasis has been linked to ageing and longevity as well. A first indication is provided by acetic acid, whose uptake by the cell induces toxicity and affects longevity. Secondly, vacuolar acidity has been linked to autophagic processes, including mitophagy. In agreement with this, a decline in vacuolar acidity was shown to induce mitochondrial dysfunction and shorten lifespan. In addition, the asymmetric inheritance of Pma1 has been associated with replicative ageing and this again links to repercussions on vacuolar pH. Taken together, accumulating evidence indicates that pH homeostasis plays a prominent role in the determination of ageing and longevity, thereby providing new perspectives and avenues to explore the underlying molecular mechanisms.

## INTRODUCTION

pH homeostasis is of crucial importance for many molecular and physiological processes. In eukaryotic cells, intracellular pH affects protein folding and enzyme activity, is required for vesicle trafficking and impacts organelle function and integrity. As our knowledge of pH homeostasis progresses, it becomes increasingly evident that the proton not only acts as a facilitator for cellular functions but also as a potent second messenger for the regulation of growth and ageing. Dysregulation of pH and lysosomal dysfunction are being linked to numerous human diseases 1, including neurodegenerative disorders 2,3. Moreover, acidity-dependent probes are used as lysosomal storage disorder-associated markers 4 and acidification of the extracellular space is known to promote cancer metastasis 5. These observations led to a growing interest in pH-related research and in this domain the unicellular organism *Saccharomyces cerevisiae *proves to be a valuable model to unravel the fundamental molecular mechanisms underlying pH homeostasis and the relationship between pH, cell growth, stress resistance and longevity.

## THE MAIN PLAYERS IN pH HOMEOSTASIS

Cytosolic and organellar pH are tightly controlled in all eukaryotic cells. The two main players of pH homeostasis in yeast are the V-ATPase and Pma1. The V-ATPase is a proton translocating ATPase that pumps protons from the cytosol into the vacuole, endosomes and Golgi compartments. Pma1 is a P-type ATPase that pumps protons over the plasma membrane to acidify the extracellular space. As accurate control of pH is required for pH homeostasis and optimal functionality of the cell and its organelles, it is not surprising that the V-ATPase and Pma1 act in close harmony. Loss of V-ATPase activity leads to a partial mislocalization of Pma1 to the vacuole and other compartments, probably reflecting a compensatory mechanism 6. In addition to the V-ATPase and Pma1, numerous other proton pumps and exchangers have been identified in yeast, and they probably fine-tune pH control of the cytoplasm and each of the organelles 7. However, given the importance of pH for organelle-specific functions, surprisingly little is known about their precise regulation and contribution to pH homeostasis.

### Regulation of the vacuolar V-ATPase

The V-ATPase is a multisubunit enzyme composed of a peripheral V_1_ sector responsible for ATP hydrolysis, and a membrane-embedded V_0_ sector responsible for proton translocation 8. In contrast to mammalian cells, which often exhibit tissue- and/or organelle-specific expression of multiple isoforms of one subunit, yeast cells only have one organelle-specific V_0_ isoform. Indeed, Vph1-containing V-ATPase complexes are localized at the vacuolar membrane, while Stv1-containing V-ATPase complexes cycle between the Golgi apparatus and endosomes 9. The activity of Vph1-containing V-ATPases is mainly regulated by reversible disassembly of the V_0_ and V_1_ sectors, in which carbon source availability plays a major role. In the presence of glucose, the sectors are assembled at the vacuolar membrane and the V-ATPase is highly active, acidifying the vacuolar lumen. When glucose is scarce, the vacuolar V_1_ sector dissociates from the V_0_ sector and the vacuolar luminal pH (pHv) becomes more alkaline 10. However, this is in no case an all-or-nothing event, as intermediate levels of assembly have been observed with varying nutrient conditions. The exact mechanism by which glucose impacts on vacuolar V-ATPase assembly remains poorly understood though several links with glucose-induced signalling events and glycolysis have been reported as further explained below. Interestingly, the vacuolar V-ATPase is also responsive to changes in cytosolic (pHc) and extracellular pH (pHe) 11,12,13. For instance, V-ATPase disassembly upon glucose-starvation is significantly reduced when cells are grown at pH 7 and accordingly, enhanced V-ATPase activity is observed in isolated vacuoles from cells grown under these conditions as compared to cells grown at pH 5 11,12. Hence, the V-ATPase appears to act as pH sensor and both in yeast and mammalian cells the vacuole-specific V_0_ subunit ‘a’, encoded by *VPH1 *in yeast, has been proposed as pH-sensing protein 11,14,15. Interestingly, this subunit also mediates the dissociation of the V_1_ and V_0_ complexes upon glucose-starvation 11. In line with this, V-ATPases that reside in the Golgi compartment, containing the *STV1*-encoded subunit ‘a’ instead of Vph1, do not dissociate upon glucose depletion 16. Besides glucose and pH, lipids also impact on V-ATPase regulation as the signalling phosphoinositides PI^3^,^5^P_2_ and PI^4^P promote V-ATPase activity at the vacuole and Golgi, respectively, through interaction with the appropriate ‘a’ subunits Vph1 and Stv1 17,18,19. Additional levels of V-ATPase regulation include the adjustment of coupling efficiency between ATP hydrolysis and proton translocation, and the mechanisms for assembly of the V_0_ complex in the endoplasmic reticulum (ER) and the subsequent export and delivery of the vacuolar V-ATPase to the vacuolar membrane 20,21,22,23,24.

### Regulation of the plasma membrane embedded Pma1

The Pma1 P-type ATPase is considered as the major determinant of pHc and plasma membrane potential in yeast 25,26. In contrast to genes encoding subunits of the V-ATPase, *PMA1 *is an essential gene, making the protein a difficult target to study. Different environmental and nutritional factors control Pma1 activity, with glucose availability being the best studied. The transcription of* PMA1* is enhanced during growth on glucose 27. In addition, the proton pump is controlled by reversible phosphorylation, which modulates an inhibitory interaction of the C-terminus with the active site of the H^+^-ATPase. The latter reflects a complex interplay of several signalling events, albeit not all players have yet been identified. Evidence obtained so far points to an involvement of the casein kinase 1 homologues Yck1 and Yck2, the protein kinase Ptk2 and the protein phosphatase Glc7 28,29,30. Calcium signalling was also shown to modulate the plasma membrane H^+^-ATPase activation in response to glucose 31. Furthermore, the intra- and extracellular pH, as well as the plasma membrane potential affect Pma1 activity. Here, an important contribution has been ascribed to the pH-dependent regulation of the potassium transporter Trk1 and the compensatory roles of K^+^ transport and H^+^ efflux to maintain the electrochemical gradient 6,32. As mentioned before, a reduction in V-ATPase activity, for instance by glucose starvation, triggers sorting of Pma1 from the Golgi to the vacuole 33.

Note that the yeast genome encodes for another plasma membrane H^+^-ATPase, i.e. Pma2, but the expression of this pump is very low and, consistently, it only has a minor impact on cellular pH 34,35.

The proton gradients established by the V-ATPase, Pma1 and other proton transport systems are of absolute importance for several processes. For instance, the driving force created by these gradients is crucial to maintain phosphate homeostasis 36 and the cation balance of alkali metals (Na^+^ and K^+^), divalent cations (Ca^2+^ and Mg^2+^) and trace metals (Fe^2+^, Zn^2+^, Cu^2+^ and Mn^2+^). Their symport/antiport along with H^+ ^helps to maintain their physiological and non-toxic levels, so as to provide a suitable environment for various biochemical reactions. The exact regulation of this cation balance has been the subject of an excellent review 7. Several membrane proteins rely on the proton driving force. Examples are the proton-coupled phosphate symporters and the different amino acid permeases that reside in the plasma membrane 36,37 or the yeast AVT1-7 family members that mediate bidirectional transport of amino acids across the vacuolar membrane 38.

## A MÉNAGE-À-TROIS FOR NUTRIENT SIGNALLING AND pH REGULATION 

As both the V-ATPase and Pma1 react closely to nutrient availability, it seems evident that proton pumping activity and pH homeostasis must be regulated by an interplay of diverse nutrient-induced signalling networks. Indeed, the protein kinases PKA and Sch9 and the protein kinase complex TORC1 play a central role. This so-called ménage-à-trois integrates input from several nutrient sensing systems in order to regulate metabolism, intracellular trafficking, proteome integrity, autophagy, stress resistance, cell size, cell cycle progression, growth and sporulation 39,40,41,42. The activity of each of these kinases is tightly regulated in response to different nutritional cues.

### Nutrient-controlled regulation of the ménage-à-trois

**Figure 1:**
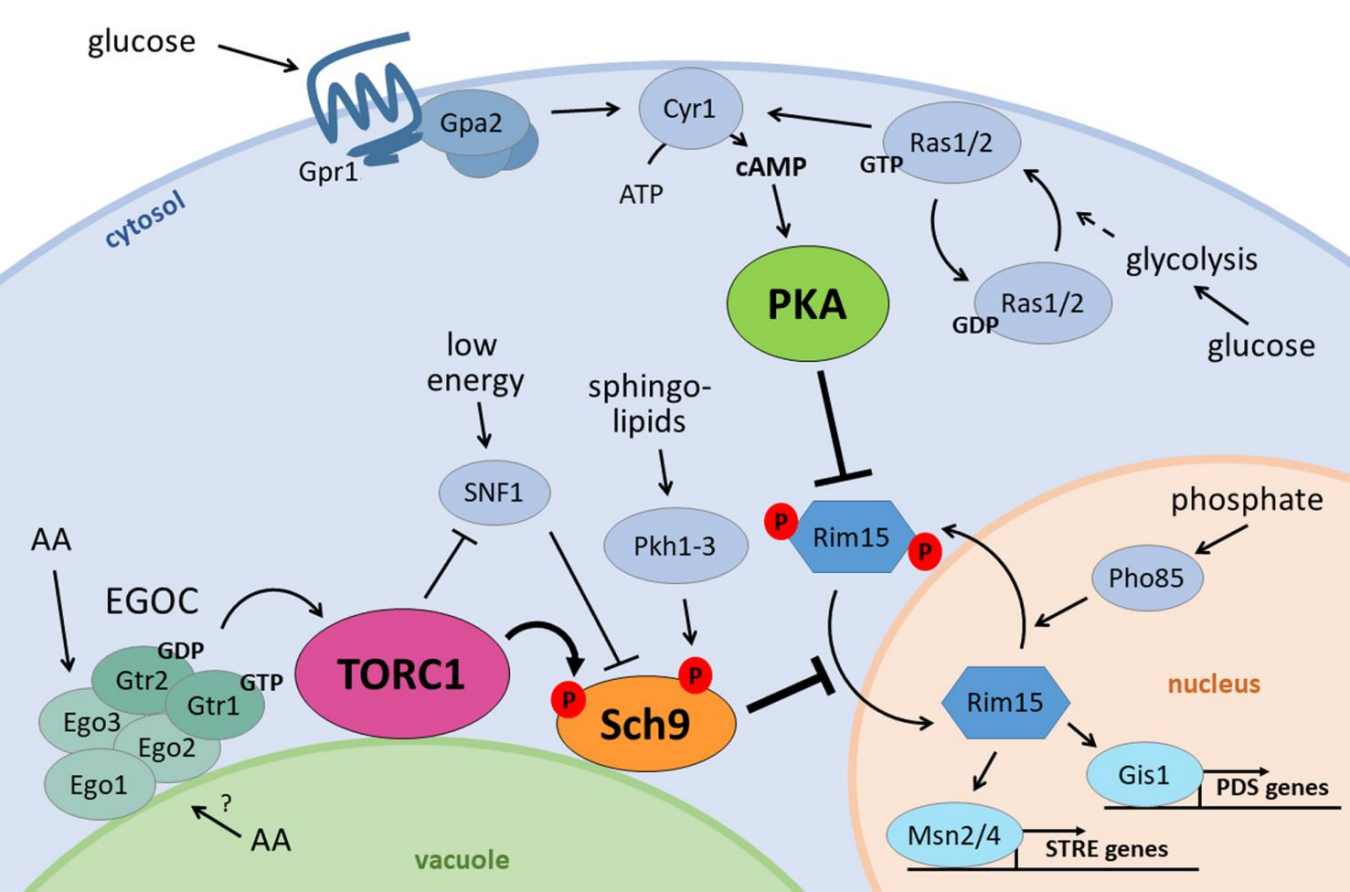
FIGURE 1: The nutrient signalling ménage-à-trois. Glucose stimulates PKA activity through the GPCR system of Gpr1 and Gpa2 on the one hand, and through activation of Ras1/2 on the other. Both induce cAMP production by Cyr1, activating PKA. At the vacuolar membrane, the EGOC senses amino acids from the cytosol and presumably the vacuole, and activates TORC1 in return. TORC1 negatively regulates the energy sensor SNF1 and activates Sch9 through phosphorylation. Besides TORC1, also SNF1 influences Sch9 activity as well as the sphingolipid effectors Pkh1-3. The PKA and TORC1/Sch9 branches converge on multiple players, perhaps the most prominent being Rim15, whose import into the nucleus is inhibited by both branches and is also controlled by phosphate availability through Pho85. When the ménage-à-trois is inactive, Rim15 can enter the nucleus where it induces transcription of PDS genes by Gis1 and STRE genes by the Msn2/4 transcription factors.

As shown in Fig. 1, the activity of PKA is regulated by the Ras-cAMP pathway and activation of adenylate cyclase, the latter depending on extracellular glucose sensing via the Gpr1/Gpa2 GPCR system as well as on intracellular glucose sensing via activation of the small G-proteins Ras1 and Ras2 43. The protein kinase complex TORC1 is regulated by intracellular amino acid signalling and this occurs by different mechanisms depending on the quality of the amino acid as a nitrogen source 44. Leucine and probably other neutral amino acids signal to TORC1 through the EGO complex (EGOC), the orthologue of the mammalian Rag-Ragulator complex. EGOC consists of Ego1, Ego2 and Ego3 that form a scaffold at the vacuolar membrane for the Rag GTPases Gtr1-2. The latter function as a heterodimer that receives input about the cytosolic amino acid content. When amino-acids are available in the cytoplasm, an active EGOC formation is triggered in which Gtr1 is in its GTP-bound form and Gtr2 in its GDP-bound form, leading to TORC1 activation. Amino acid starvation induces the opposite GTP/GDP-loading status of the GTPases and the consequent inhibition of TORC1 45,46. Recent evidence obtained in mammalian systems suggests a role for the Rag-Ragulator in signalling lysosomal amino acid content and transport in addition to cytosolic amino acid content 47,48, but whether EGOC has a similar role in sensing the vacuolar amino acid load in yeast remains to be studied. Preferred nitrogen sources like glutamine can signal independently of EGOC to trigger a more sustained TORC1 activation required to support vigorous growth 44. At present, the underlying mechanism for this Gtr1/2-independent TORC1 activation in yeast remains poorly understood, though one study identified Ypt1, a GTPase involved in ER-to-Golgi vesicular trafficking, as an alternative regulator of TORC1 49. Other studies suggested a role for the vacuolar membrane-associated phosphatidylinositol (PI) 3-phosphate binding protein Pib2 and the Vps15/34 Pl 3-kinase complex 50,51. The third member of the ménage-à-trois, the AGC kinase Sch9, is the yeast orthologue of mammalian PKB/Akt and S6 kinase. It is a well-known target of TORC1 and as such it is involved in amino acid and nitrogen source signalling 52. However, Sch9 has been implicated in the sensing and signalling of other nutrients as well. It plays a major role in lipid signalling as a regulator of the so-called sphingolipid rheostat 53 and, besides its phosphorylation by TORC1, Sch9 needs to be phosphorylated by sphingolipid-activated kinases Pkh1-3 at its PDK1 site in the activation loop to obtain full activity 52,54,55. Moreover, Sch9 also responds to glucose availability, thereby acting in conjunction with PKA 56, and receives input of the protein kinase complex SNF1, a central regulator of cellular energy homeostasis 57. Thus, Sch9 appears to play an integratory role in nutrient sensing that allows for coordination and fine-tuning of diverse signalling cascades. As such, it shares several effectors with the PKA and TORC1 signalling routes but for several of these targets the effect inflicted by Sch9 can either be additive or opposite depending on the activity status of the other kinases 41,58,59,60.

An example of the interplay between the signalling pathways controlled by the ménage-a-trois is their convergence to regulate the activity and nuclear localization of Rim15, a protein kinase required for metabolic adaptation and the general stress response (Fig. 1) 61. Here, also phosphate availability comes into the picture as the nuclear exit of Rim15 is controlled by the phosphate-responsive PHO-pathway, particularly Pho85 41,62.

### The nutrient status regulates pH homeostasis

The most straightforward evidence for the role of nutrient signalling in pH regulation are the alterations of extra- and intracellular pH in response to nutrient availability and growth rate. Albeit quite complex, we can summarize this as follows. When cells grow fermentatively, they produce organic acids that acidify the medium, such as acetic acid. In its protonated form, acetic acid is able to move through the plasma membrane back into the cell 63. Once inside the cytosol, whose pH is maintained around neutrality during exponential growth, protons dissociate from the weak acid causing intracellular acidification. This acidification is counteracted by Pma1-mediated proton efflux and V-ATPase-mediated vacuolar acidification in order to maintain pHc homeostasis 9,64,65,66. During the diauxic shift or upon glucose starvation, the activity of Pma1 reduces, the cytosol acidifies and the vacuolar V-ATPase disassembles 11,67,68,69,70. Re-addition of glucose or sucrose to glucose-starved cells initially results in a rapid drop in pHc, probably due to the resumption of glycolysis, but this is quickly followed by intracellular alkalization and extracellular acidification as a result of Pma1 and vacuolar V-ATPases regaining full activity 11,70,71,72. In contrast to the clear impact of carbon source availability, re-addition of amino acids or another nitrogen source to starved cells does not affect pHc 70,73. This demonstrates that adjusting pHc is not simply a matter of growth resumption, but that it is specifically controlled by carbon source availability.

### The ménage-à-trois is crucial for pH regulation

**Figure 2:**
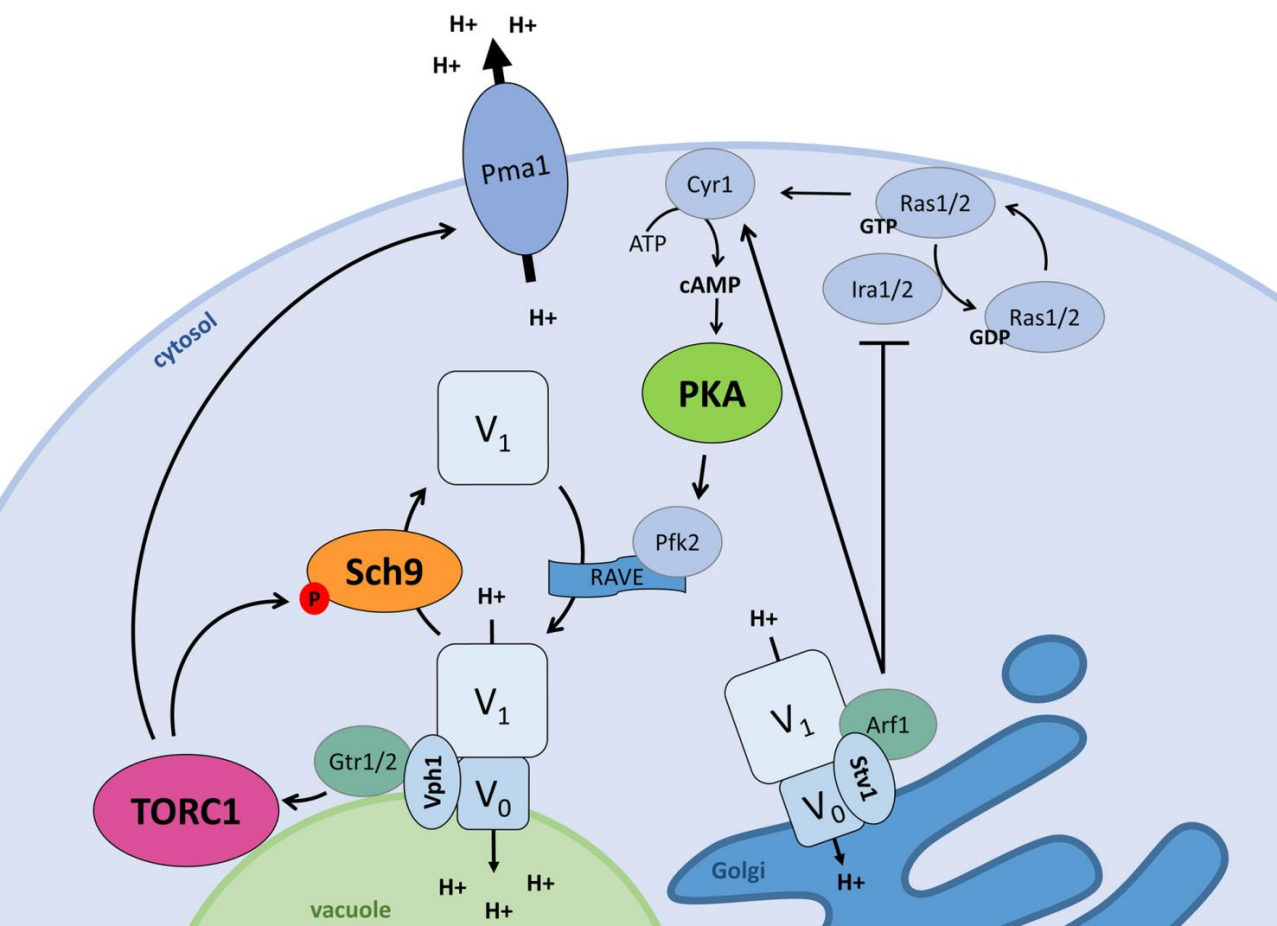
FIGURE 2: The interplay between signalling and pH control. Pma1 and the V-ATPase, the two main players in pH control, both impact and are regulated by the ménage-à-trois. When stimulated by glucose or cytosolic protons, the vacuolar Vph1-containing V-ATPase stimulates TORC1 via the EGOC-components Gtr1/2. The Golgi-specific Stv1-containing V-ATPase stimulates PKA via Ira1/2 inhibition by Arf1, pushing Ras1/2 towards the GTP-bound state and in turn activating Cyr1 to produce cAMP. Active TORC1 and PKA each regulate the vacuolar V-ATPase assembly state. TORC1 stimulates disassembly via Sch9, while PKA aids V-ATPase assembly presumably via Pfk2 which impacts on the V-ATPase scaffolding complex RAVE. Additionally, the vacuolar V-ATPase subunit Vph1 acts as a proton sensor to induce V-ATPase activity, and this may also be the case for the Stv1-containing V-ATPase. Additionally, active TORC1 also stimulates Pma1 activity.

The ménage-à-trois plays an important role to integrate nutrient availability and pH homeostasis as shown in Fig. 2. Alterations that enhance PKA activity, like deletion of the Ras2 GTPase *IRA2* or the PKA regulatory subunit* BCY1*, were found to inhibit vacuolar V-ATPase disassembly upon glucose deprivation. This suggests that PKA is involved in glucose-dependent V-ATPase regulation 74. Although PKA-dependent phosphorylation has been described for the V_1_ subunit ‘C’ in insect cells 75 and for the V_1_ subunit ‘A’ in human HEK-293T cells 76, no such event has been reported in yeast so far. Nonetheless, the Ras-cAMP pathway and PKA are known to regulate key enzymes of glycolysis in yeast 41,77 and two glycolytic enzymes, i.e. aldolase and phosphofructokinase, were reported to associate with the V-ATPase, placing them in pole position to mediate the glucose signal 67,78,79,80. Especially the phosphofructokinase Pfk2 might be a good candidate as it is a direct target of PKA 81. Pfk2 is indeed required to maintain vacuolar acidification and optimal RAVE-mediated reassembly of the V_0_ and V_1_ subunits upon re-addition of glucose to cells that were briefly deprived of the sugar 67,78. RAVE is the acronym for ‘Regulator of H^+^-ATPase of Vacuolar and Endosomal membranes’ and is a complex known to function as a scaffold that binds the V_0_ and V_1_ sectors in a glucose-dependent manner 82,83. Earlier studies also suggested a possible role for the Ras-cAMP pathway in the glucose-induced activation of Pma1 84,85. However, subsequent detailed analysis contradicted such a role and showed that the plasma membrane H^+^-ATPase is still activated in mutant strains deficient for glucose-induced cAMP increase and that similar activation levels are obtained in strains with normal or attenuated PKA activity 86,87.

Although starvation and resupplementation of nitrogen and amino acids does not influence pHc 70,73, both TORC1 and Sch9 are involved in the regulation of pH homeostasis in function of glucose availability. When cells are grown on glucose both players are localized at the vacuolar membrane, but once cells enter the diauxic shift, Sch9 is displaced into the cytosol. The latter occurs concomitantly with the disassembly of the vacuolar V-ATPase 70. In fact, Sch9 has a modulatory role in controlling the V-ATPase assembly state as evidenced by the observation that cells lacking Sch9 display an enhanced association of the V_0_ and V_1_ sectors in the presence of glucose and a delayed disassembly of the proton pump upon glucose starvation. Similar effects were also seen upon rapamycin-induced TORC1 inhibition or upon replacement of the wild type Sch9 allele by the Sch9^5A^ version that can no longer be activated by TORC1 70. Accordingly, *sch9*∆ cells have more acidic vacuoles both in the presence or absence of glucose, and while *sch9*∆ mutant cells are able to maintain their pHc within the same range as wild type (WT) cells during fermentative growth, once they traverse the diauxic shift a hyperacidification of the cytosol is observed 70. Whether the latter points to a role of Sch9 in the regulation of Pma1 or other ion pumps and exchangers remains to be investigated in more detail, but at least two observations suggest that this may well be the case. Indeed, *sch9*∆ cells fail to rapidly acidify the extracellular medium when glucose is refed to glucose-starved cells and the additional deletion of *SCH9* in strains lacking a functional V-ATPase triggers a further alkalization of the vacuolar lumen 70. Interestingly, a most recent paper reported that TORC1 is required to obtain full Pma1 activity and although a possible role of Sch9 was not examined, the study demonstrated an involvement of the protein phosphatase Sit4, another TORC1 effector, in the turnover but not the phosphorylation status of Pma1. The same study also showed that both *sit4*∆ cells and cells lacking the TORC1 subunit Tco89 display reduced K^+^ uptake and a more acidic intracellular pH as compared to WT cells, but solely in the *sit4*∆ mutant this was associated with a reduced Pma1 activity 88.

### Additional players link nutrient signalling with pH homeostasis

Apart from the effects mediated by the PKA and TORC1-Sch9 axis on V-ATPase assembly/disassembly or Pma1 activity, other players involved in nutrient signalling also affect pH homeostasis. As mentioned, the signalling lipid PI^3^,^5^P_2_ was shown to be important for pHv regulation. Accordingly, the PI^3^,^5^P_2_-deficient mutants *vac7*, *vac14 *and *fab1* all display vacuolar acidification defects 89,90,91. Although PI^3^,^5^P_2_ may directly affect V-ATPase activity 18, the phosphoinositide may also act indirectly via TORC1/Sch9 signalling, as TORC1 activity as well as the recruitment of Sch9 and its TORC1-dependent phosphorylation at the vacuolar membrane are dependent on the presence of PI^3^,^5^P_2_ 92,93,94. Moreover, increased PI^3^,^5^P_2_ levels are associated with vacuolar fragmentation, which involves TORC1 and its downstream effectors 95,96. This relationship is further highlighted by the significant overlap in targets between a genome-wide screening aiming to identify genes conferring a synthetic sick/lethal phenotype when combined with the *SCH9* deletion and a screening for genes mediating inositol auxotrophy 70,97. Similar genome-wide screenings were performed to find proteins involved in the control of pHc or pHv during fermentative growth on glucose 24,25 and at least with the screening on pHc a significant overlap is seen when compared to the data of the synthetic *SCH9* screening 70. Besides proteins associated with vesicular transport, lipid metabolism or amino acid biosynthesis, both pH screenings retrieved proteins required formitochondrial functions. This is intriguing because it indicates that mitochondria are essential to maintain pH homeostasis even during fermentative growth, where they are not required for energy supply. This underscores the importance of cross-talk between organelles, and the notable lack of research performed to date in this area.

### pH as a second messenger for nutrient signalling

Changes in intracellular pH affect cell functioning at different levels as it impacts on protein folding, enzyme activities and the protonation of biological macromolecules, lipids and other metabolites 66. The crucial question, however, is whether alterations in pH are sensed and perceived as signals. Perhaps the best way to answer this is to describe the recent advances in how pH affects the activities of the PKA/TORC1/Sch9 ménage-à-trois.

Already decades ago, it was reported that the treatment of fungi with depolarizing agents inflict a rapid increase in the level of cAMP 98,99,100. Mechanistically, different scenarios were described as intracellular acidification was found to enhance both the affinity of the adenylate cyclase Cyr1 for its substrate ATP 98 as well as Ras-GTP loading by inhibition of the GTPase-activating proteins, Ira1 and Ira2 101. Notably, a mild treatment of cells with a protonophore allows to bypass the requirement of hexose transport for glucose-induced activation of the Ras-cAMP pathway 102, which is consistent with the observation that the protonophore treatment of glucose-starved cells triggers a rapid drop in pHc similar as that seen immediately after addition of glucose to these starved cells 98. A more recent study correlated the glucose-induced changes in pHc to V-ATPase assembly/disassembly and subsequent activation of PKA 11. As mentioned, this study also proposed the V ATPase to act as pH-sensor with Vph1, the V_0_ subunit ‘a’, as putative pH-sensing protein. Subsequently, the role of the V-ATPase for activation of the Ras-cAMP pathway was further defined, since in the presence of glucose the pump was shown to signal to the Ras proteins via Arf1, a GTPase that interacts with the Golgi-specific V_0_ subunit Stv1. Moreover, the activity of the plasma membrane ATPase Pma1 was shown to stimulate growth through Ras activation by increasing pHc 73.

Additionally, pHc and the V-ATPase also confer signals for glucose availability to TORC1 and Sch9. To this end, the V-ATPase controls the GTP-load of Gtr1 and interacts with this EGOC Rag GTPase via the vacuole-specific V_0_ subunit Vph1. This finding is rather remarkable as Gtr1 plays a pivotal role in amino acid sensing 45,46, but amino acid availability itself does not affect pHc nor the V-ATPase assembly state 70,73. As the V-ATPase apparently acts as activator of both PKA and the TORC1-Sch9 axis, while each of the kinases has the ability to provide feedback by affecting the V-ATPase assembly/disassembly, a very balanced system based on feedback loops is established (Fig. 2). The necessity of this tight control relates undoubtedly to the crucial roles of the PKA/TORC1/Sch9 ménage-à-trois for the overall cellular functioning and the coordination between growth and the cell cycle 41. The latter is further evidenced by the finding that the TORC1-Sch9 axis transmits signals from the vacuole that are required for cell cycle progression 94.

A signalling role of pHc also became apparent from data obtained from a genome-wide screening that investigated the correlation between aberrant intracellular pH and reduced growth rate of mutants. This analysis confirmed a tight connection between both, suggesting that pHc dictates the growth rate. For 19 out of the 173 mutants, however, the causal relationship between pHc and growth rate was completely abrogated, indicative that these mutants fail to properly sense the pH signal. Among them were mutants affected in mitochondrial translation, inositol phosphate biosynthesis and lipid biosynthesis 25. Importantly, a similar study found no causal correlation between pHv and growth rate 24.

## pH CONTROL, STRESS TOLERANCE AND LONGEVITY 

**Figure 3:**
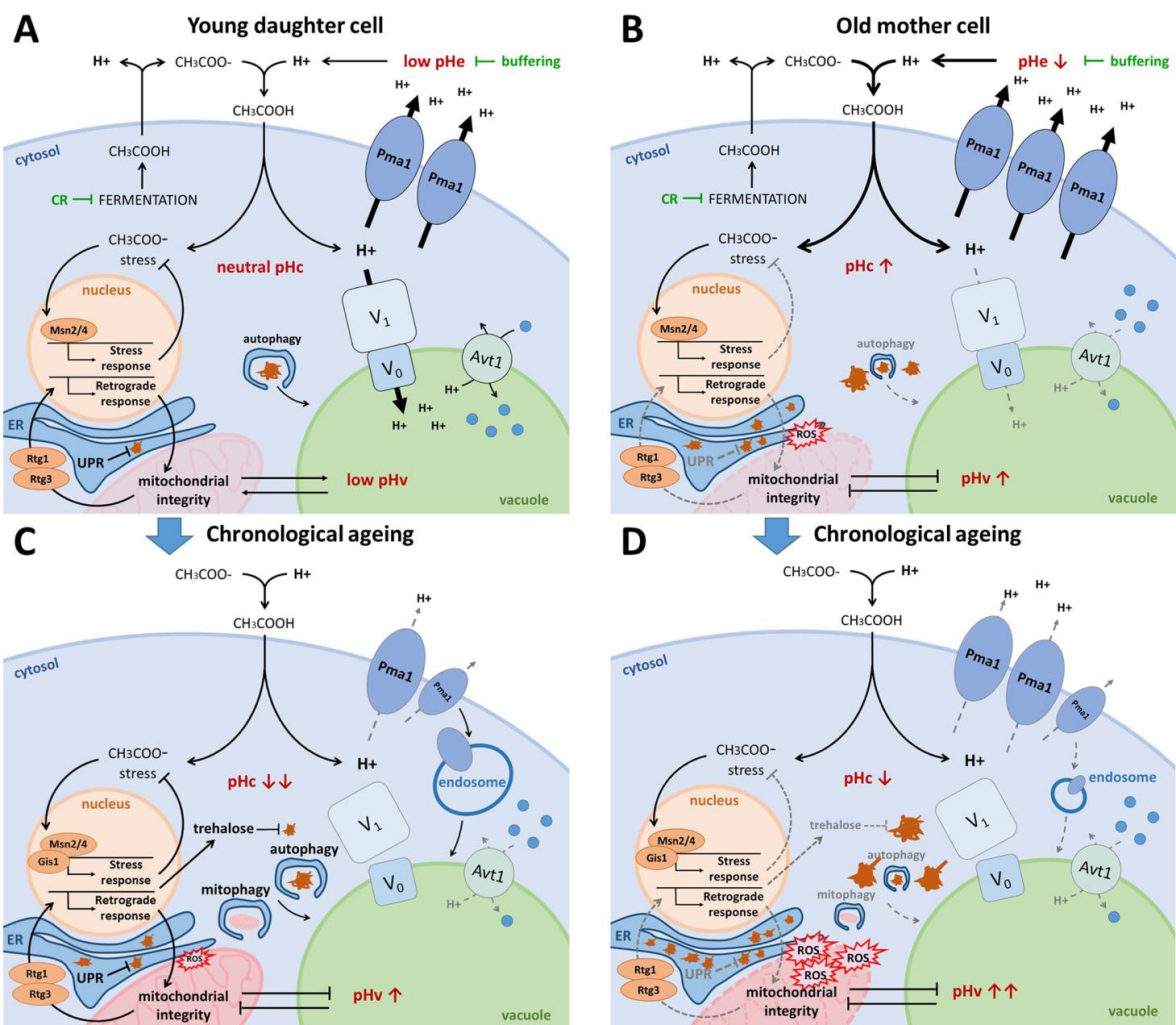
FIGURE 3: The interplay between pH control and ageing. **(A)** **Young daughter cell.** Fermentatively growing cells produce acetic acid, which is extruded from the cell. When protonated in the acidic medium, acetic acid passes the plasma membrane causing cytosolic acidification and acetic acid stress. CR and medium buffering counteract this phenomenon by reducing fermentation and protonation of acetic acid respectively. The optimally active Pma1 and V-ATPase retain the cytosolic pH around neutrality by counteracting this proton influx. The resulting acidic vacuole allows for optimal amino acid uptake by the proton/amino acid antiporter Avt1 and efficient autophagy. Acetic acid stress induces the environmental stress response aided by transcription factors Msn2/4. Albeit mitochondria are not required for energy production on fermentative medium, they do contribute to the maintenance of pH homeostasis. The retrograde response, mediated by Rtg1/3, assures mitochondrial integrity. In the event of protein misfolding, the UPR is activated in the ER. Damaged and aggregated proteins are efficiently cleared by autophagy and the ubiquitin-proteasome system (UPS; not shown). **(B)** **Old mother cell.** Due to asymmetrical inheritance, mother cells possess an abundance of Pma1 proteins, presumably raising the cytosolic pH, which likely also causes an unavailability of protons to be traversed to the vacuole. The resulting vacuolar alkalization causes insufficient amino acid import by Avt1, leading to mitochondrial dysfunction by a yet unknown mechanism. The retrograde response is activated in an attempt to counteract mitochondrial dysfunction, though it may not be sufficient to maintain mitochondrial integrity. Misfolded proteins accumulate in the ER thereby triggering ER stress, and damaged proteins accumulate in the cytosol due to impaired autophagy and an overwhelmed UPS (not shown). Both reduced mitochondrial integrity and protein misfolding lead to the formation of reactive oxygen species (ROS) and they affect lipid homeostasis thereby further impairing vacuolar functioning. **(C)** **Chronological ageing of young daughter cell.** When traversing the diauxic shift, cells experience a disassembly of the V-ATPase and a reduction in Pma1 activity, aided by its endocytosis. As a result, the cytosol acidifies and the vacuole alkalizes. This presumably, similar as in replicatively ageing cells, leads to an impairment of vacuolar amino acid uptake, autophagy, mitophagy and endocytosis. In post-diauxic shift cells, Msn2/4 and Gis1 are activated to induce stress responses and the production of trehalose, which helps to protect cells from acetic acid stress and toxicity of damaged proteins. Although stress responses are optimal in young daughter cells traversing post-diauxic shift, intracellular damage will accumulate over time, eventually surpassing the capacity of the protective machinery, leading to cell demise. **(D) Hypothetical model of chronological ageing in old mother cell.** In this model we assume a cumulative deregulation of the already decontrolled pH homeostasis in old mother cells following post-diauxic shift. This should lead to an additional impairment of vacuolar amino acid uptake, autophagy, mitophagy and endocytosis and cause a quick accumulation of intracellular damage, leading to more rapid cell demise.

It is well established that the nutrient pathways controlled by the PKA/TORC1/Sch9 ménage-à-trois have a significant impact on cellular ageing, whether being monitored as replicative lifespan (RLS) by assessing the number of divisions a mother cell can undergo before dying, or as chronological lifespan (CLS) by assessing the time span a non-dividing cell remains viable 103. Both modes of ageing rely on partially overlapping cellular and molecular determinants and these have been the topic of excellent reviews to which we refer for more details 40,104,105,106,107,108,109,110,111,112. As nutrient availability and the PKA/TORC1/Sch9 ménage-à-trois affect pH control, it is not surprising that pH is being linked to the regulation of ageing/longevity in yeast. Hence, a number of groups have explored the interplay between extracellular, cytosolic and organelle pH and longevity, the topic of the sections below and summarized in Fig. 3.

### Acetic acid shortens longevity and induces programmed cell death

A nice example that illustrates the interconnections between lifespan, nutrient availability and their dependence on pH is the observation that chronologically aged cells display a reduced subsequent RLS, but that this can be attenuated when the chronological ageing occurs in buffered medium or in calorie restriction (CR) conditions 113,114. As mentioned, during fermentative growth yeast cells produce and secrete different organic acids. While some of these acids accumulate in the growth medium, acetic acid levels decline after post-diauxic shift, indicating that this acid is used as a carbon source. The drawback is, however, that acetic acid uptake by the cell comes with a degree of toxicity that affects longevity (Fig. 3). Consistently, growing cells under acidic conditions was found to shorten CLS and RLS, while CR significantly reduces the production of organic acids and extends CLS and RLS 114,115,116. Buffering the medium also has a positive effect on lifespan as it lowers the difference between the extra- and intracellular pH values, thereby reducing the driving force for inwards diffusion of acetic acid. As such, buffering presumably prevents stress by reducing the amount of energy that needs to be consumed to maintain the intracellular pH 117.

Indeed, as a first line of defense against acetic-acid induced stress, cells rely on energy-consuming proton pumps to counteract cytosolic acidification caused by dissociation of protons from the acetic acid once it has entered the cell, and to maintain pH optima in all different cellular compartments. Here, the plasma membrane-embedded Pma1 fulfils an important role by extruding these protons from the cytosol, allowing healthy exponential WT cells to maintain pHc around neutrality independently of pHe (Fig. 3A) 71. During ageing however, yeast cells struggle to maintain proper pH homeostasis. As mother cells undergo divisions, the pHc increases whereas daughter cells retain a more acidic cytosol (Fig. 3B). This phenomenon is attributed to asymmetrical distribution of Pma1, which predominantly remains in the plasma membrane of the mother cell 118. This mother-specific increase in Pma1 activity and subsequent reduction of cytosolic proton content is believed to trigger the decline of vacuolar acidity during replicative ageing, as protons are unavailable for the V-ATPase to be pumped into the vacuolar lumen 118. Conversely, when cells have traversed the diauxic shift or when encountering glucose depletion, a condition typically used to study chronological ageing, the activity of Pma1 declines as mentioned previously. Moreover, also the V-ATPase is disassembled under these conditions 11,67,68,69,70 and this loss of V-ATPase activity signals ubiquitination and endocytosis of Pma1 119. In consequence, the pHc is significantly reduced both in post-diauxic cells and in chronologically aged cells and due to the lower activity of the V-ATPase, the vacuole presumably becomes more alkaline (Fig. 3C).

Secondly, mild stress induces the so-called general or environmental stress response pathway, an adaptive response that allows to acquire a form of stress resistance that protects cells from subsequent stress triggered by the same or another stressor 120,121. This adaptive response applies to acetic acid-induced stress as well as to physiological stresses affecting lifespan 122 and it is the basis of hormesis effects that play in ageing 123,124. In the environmental stress response pathway, the transcription factors Msn2/4 play a central role. Msn2/4 are well known targets of the PKA/TORC1/Sch9 ménage-à-trois required to induce expression of several stress-responsive genes needed to protect cells from adverse conditions (Fig. 1; Fig. 3) 41. Notably, these transcription factors are also induced when cells encounter alkaline stress 125,126, suggesting that the actual stress factor might be a change in pH, the proton gradient or membrane potential. A study that aimed to identify genes essential for the acquisition of tolerance to different weak acids implicated Msn2/4 for acetic acid tolerance 127. Interestingly, apart from vacuolar acidification, intracellular trafficking and ergosterol biosynthesis, acetic acid tolerance was found to specifically depend on a small set of genes and this included those encoding Ras2, the trehalose-6P synthase, different cytosolic and mitochondrial ribosomal proteins and two well-known players of the retrograde response pathway, i.e. Rtg2 and Rtg3 127. These data nicely complement observations connecting metabolism, cell death and longevity. The finding of mitochondrial ribosomal proteins and components of the retrograde response pathway is consistent with the requirement of functional mitochondria to maintain pH homeostasis 24,25, and there is ample evidence that mitochondrial dysfunction accompanies acetic acid-induced programmed cell death (PCD) 128,129 and the reduction of CLS and RLS (Fig. 3B-D) 130,131,132,133,134,135. Upon mitochondrial dysfunction, the retrograde response pathway is triggered to transmit signals to the nucleus in order to make adjustments in cellular metabolic and biosynthetic activities. The retrograde response pathway is positively controlled by Ras2, explaining why this small GTPase is essential for the acquisition of acetic acid tolerance. In fact, PKA, similar as TORC1, negatively influences the retrograde response pathway 136. Among other genes, the retrograde response pathway targets several cytosolic and mitochondrial ribosomal protein genes 137, explaining their involvement in acetic acid tolerance.

Some evidence suggests that the expression of genes involved in trehalose biosynthesis is also influenced by the retrograde response pathway 137. Although the role of trehalose in conveying acetic acid tolerance is not fully understood, the long-lived *tor1*∆ and *sch9*∆ mutants appear to make optimal use of the protective properties of trehalose as these strains were found to switch their metabolism in the quiescent phase and use the acetic acid that was secreted during the pre-diauxic phase to produce trehalose 138. A recent study reported that Tps1 can decelerate chronological ageing independently of its known trehalose-6P catalytic activity 139,140. Consistently, the *tps1*∆ mutant is short-lived. However, another study demonstrated that this phenotype is shared by mutants lacking the trehalose-6P phosphatase Tps2, or the neutral trehalases Nth1 and Nth2. In contrast, mutants lacking the regulatory subunits of the trehalose synthase complex Tps3 or Tsl1, or the periplasmic acid trehalase Ath1, were found to be long-lived 141. Analysis of these different mutants revealed that trehalose reduces the amount of oxidative carbonylated proteins during post-diauxic phase and that it lowers the level of protein aggregation in the quiescent state 141. Thus, trehalose seems to protect cells by preventing proteotoxicity (Fig. 3C-D).

### pH and proteotoxicity

Cells are equipped with a complex network that ensures proteome integrity. Proteins that can no longer fulfil their function due to misfolding, damage or aggregation become substrates for the protein degradation machinery and they are cleared either via the proteasome, or via autophagy and vacuolar targeting (Fig. 3). Here the link to pH homeostasis is obvious, as the final step of autophagy, i.e. the disintegration of autophagic bodies, is linked to vacuolar membrane integrity and acidification of the vacuolar lumen by the V-ATPase 142,143,144. Next to its detoxifying function, however, autophagy also has a role in nutrient recycling. In that regard, it seems logical that autophagy is upregulated in nutrient limiting conditions and that the nutrient signalling ménage-à-trois plays an important role in the regulation of autophagy 40,41,60. Interestingly, the cellular capacity for autophagic degradation declines with age, which will itself also contribute to the accumulation of cellular and molecular damage. Accordingly, induction of autophagy extends lifespan, and this seems to be accompanied by vacuolar acidification (Fig. 3) 143,145.

Recent evidence indicates that misfolded and damaged proteins are first partitioned in specific inclusions in the cell. In JunQ and INQ misfolded proteins are refolded, while those deposited in IPOD probably await clearance via autophagy 146,147. This partitioning requires Hsp104 and functional actin cables 148,149,150, the latter being another essential lifespan determinant 151,152. Partitioning of misfolded proteins is beneficial during chronological ageing 153 and assures the asymmetric inheritance of damaged and non-functional proteins during replicative ageing, thereby producing rejuvenated daughter cells 148,149,150. A recent study connected the V-ATPase, vesicular trafficking and components involved in actin cable-dependent vacuole inheritance to this asymmetric inheritance 154, implying a pH dependency. Notably, apart from the vacuole also the ER, mitochondria and even mRNA are subject to asymmetric inheritance during the division of yeast cells (reviewed in 155).

### Mitochondrial dysfunction and its interplay with the ER and the vacuole

Several molecular chaperones assist in the correct folding of proteins or the refolding in case protein misfolding occurs. These chaperone proteins can be found in the cytoplasm, the ER and mitochondria. In fact, a recent study revealed that yeast cells possess a cell-wide proteostasis system where proteotoxicity in one cellular compartment triggers a response in other compartments. This response, termed cross-organelle stress response (CORE) has a protective role and extends both CLS and RLS 156. Aberrant chaperone activity in each of the compartments leads to fragmented mitochondria, a loss of respiratory activity and an increase in cytosolic NADPH reducing power. This effect is associated with inactivation of TORC1, which acts as protein folding sensor, and the subsequent activation of Snf1 156,157. The existence of a cell-wide proteostasis system is also inferred by the observation that yeast cells use a common system to monitor and ensure protein quality control in the ER and mitochondria. This system involves Cdc48/p97, an AAA ATPase known from the ubiquitin-proteasome system that is recruited by stressed ER or mitochondria to extract ubiquitinated proteins presented at the membranes of the organelles and to direct these to the proteasome 158,159. Interestingly, several physical organelle contact sites exist in yeast, although their involvement in CORE and the cell-wide proteostasis system remains to be elucidated. Here we mention ERMES, the ER-mitochondrial contact site 160, and vCLAMP, the vacuole-mitochondrion contact site 161. Several nutrient and ion transporters have been shown to be enriched at these contact sites, indicating a role as hubs for the exchange of nutrients and ions between organelles 161 and pointing towards the idea that pH could be an essential regulator. In addition, ERMES and vCLAMP have been proposed to serve as dynamic metabolic signalling hubs 162. Notably, they are co-regulated in response to nutrients and appear to fulfil partially overlapping functions as their simultaneous disruption is lethal 161.

It is obvious that mitochondria play an important role in the determination of lifespan that surpasses their function as energy supplying factory. It is essential for viability that dysfunctional mitochondria are removed, which occurs via mitophagy 40. Efficient mitophagy requires ubiquitination of the ERMES components Mdm34 and Mdm12 163. Moreover, ERMES colocalizes with the site of mitophagosome generation and the ER was proposed to deliver the necessary lipids for membrane engulfment of the mitochondrion 164,165. Whether vCLAMP has a role in mitophagy has not been investigated yet. Nonetheless, it is known that it suffices to enhance vacuolar proton pumping to significantly reduce mitochondrial dysfunction, indicating a communication between these organelles which could rely on vCLAMP. Consistently, cells lacking functional V-ATPases display significant increased levels of markers for dysfunctional mitochondria and a dramatically shortened RLS and CLS (Fig. 3B-D) 70,134. Intriguingly, this appears not to be related to a reduced capacity of vacuolar protein degradation, but rather to the impaired ability of the vacuole to efficiently store amino acids. This is evidenced by the observation that enhanced expression of the neutral amino acid transporter Avt1 attenuates mitochondrial dysfunction in replicative ageing cells without preventing vacuolar alkalization 134. How this relates to the regulation of autophagic processes by the PKA/TORC1/Sch9 ménage-à-trois 40,166,167 remains to be investigated.

### pH, lipid synthesis and liponecrosis

The synthesis of major membrane lipids is spatially organized and involves different organelles 168. Moreover, many lipid synthesizing enzymes are enriched at the contact sites between the ER and other organelles and these contacts mediate non-vesicular selective lipid transport 169,170,171,172,173,174,175. As organelles rely on pH for optimal functionality, it is expected that compartmental cross-talk for lipid metabolism is closely connected to pH regulation. This is supported by the observation that membrane contact sites constitute domains important for ion transport as well 161,176, which in case of the ER-plasma membrane contact is linked to proton pumping by Pma1 177. Moreover, a study that used a systems biology approach to investigate the interdependence of pH control and CLS by comparison of young and old cells grown in buffered or non-buffered medium revealed that pH has a main impact on the reorganization of lipid metabolism. This reorganization has a beneficial effect on CLS by preserving mitochondrial and vacuolar health, the latter being dependent on V-ATPase activity 178.

Lipid homeostasis is of utmost importance to maintain longevity of yeast cells and persistent deviations thereof can lead to cell demise and a phenomenon described as liponecrosis 105,179,180,181. Several observations indicate that alterations in lipid homeostasis induce mechanisms involved in protein quality control. Conditions that perturb lipid biosynthesis, that alter the lipid compositions of the plasma membrane and endomembranes or that affect the lipid droplet content of cells were all reported to trigger ER stress and activation of the unfolded protein response (UPR) 182,183,184,185,186,187,188. In contrast to acute ER stress, which induces PCD 189,190, the lipid-associated induction of ER stress appears to be linked to compensatory mechanisms directed to reinstate lipid biosynthesis and lipid metabolism and to promote cell survival 191,192,193,194,195. For instance, the activation of the UPR was shown to restore normal ceramide levels when sphingolipid biosynthesis was compromised 192 and to enhance synthesis of triacylglycerols and sterol esters in order to stimulate the formation of lipid droplets 196. Besides their role for energy storage, these lipid droplets are essential for the regulation of autophagic processes and the clearance of damaged and aggregated proteins from the ER and mitochondria 197,198,199,200,201. Interventions that affect lipid homeostasis are commonly associated with the appearance of fragmented vacuoles and V-ATPase dysfunction. This includes alterations in the biosynthesis of ergosterols, sphingolipids and ceramides or the availability of essential precursors like inositol 202,203,204,205. However, preventing lipid droplet formation by blocking the synthesis of di- and triacylglycerol through deletion of the *PAH1*-encoded phosphatidic acid phosphatase appears to be an exception since *pah1*∆ cells are characterized by vacuolar fragmentation and enhanced lipid toxicity while still displaying improved acidification of the vacuolar lumen due to elevated expression of V-ATPase subunits 206,207. The latter relates to a negative effect of Pah1 on the transcription of several V-ATPase subunit genes, which all contain an UAS*_INO_* element in their promotor 206. This is interesting since this promotor element also links the expression of these V-ATPase genes to phospholipid biosynthesis and the availability of inositol, choline and phosphate 208.

## CONCLUDING REMARKS

Taken together, control of pH homeostasis is emerging as a key factor determining longevity and alterations culminate in many hallmarks of ageing. Although we are only beginning to uncover the importance of pH homeostasis, it is already amazing how many aspects of cell functioning are influenced by intracellular pH and the reciprocal regulation of mainly two players, the V-ATPase and Pma1. When thriving in an environment with plentiful nutrients, Pma1 and the V-ATPase maintain an ideal pH to support vigorous cell growth. During ageing, the activity of these two players changes, and while increased Pma1 activity may provide enhanced tolerance of a mother cell to the weak acids that were produced, the drawback is a reduction of vacuolar acidity. Indeed, vacuolar acidity is of absolute importance for endocytosis and vesicular trafficking, as well as cellular damage control, the latter via the clearance of malfunctioning proteins and organelles, important for both CLS and RLS (Fig. 3). In addition, damage control also occurs via asymmetrical inheritance during replicative ageing and also here links with vacuolar functioning and pHv are emerging. Undoubtedly, this field of research opens a promising path towards the understanding of intrinsic mechanisms of ageing and longevity, which could be of critical value for our insight into lysosomal-related human diseases and proteopathies, including neurodegenerative disorders, different types of cancer and lysosomal storage diseases.
